# The importance of genetic testing in the diagnosis and management of peripartum cardiomyopathy: a case study

**DOI:** 10.1093/eschf/xvag090

**Published:** 2026-04-01

**Authors:** Zoee U D’Costa, Eugene K Wong, Min Zhang, Gregory A Fishbein, Jessica Wang, Megan Kamath, Negeen Shahandeh

**Affiliations:** Department of Medicine, UCLA, Los Angeles, 757 Westwood Plaza, Los Angeles, CA 90095, USA; Department of Human Genetics, UCLA, Los Angeles, CA, USA; Department of Pathology and Laboratory Medicine, David Geffen School of Medicine, UCLA, Los Angeles, CA, USA; Department of Pathology and Laboratory Medicine, David Geffen School of Medicine, UCLA, Los Angeles, CA, USA; Department of Medicine, Division of Cardiology, UCLA, Los Angeles, CA, USA; Department of Medicine, Division of Cardiology, UCLA, Los Angeles, CA, USA; Department of Medicine, Division of Cardiology, UCLA, Los Angeles, CA, USA

**Keywords:** Peripartum cardiomyopathy, Arrhythmogenic cardiomyopathy, Genetic testing, VA-ECMO, Desmoplakin

## Introduction

Up to 20% of women diagnosed with peripartum cardiomyopathy (PPCM) carry pathogenic genetic variants associated with dilated or arrhythmogenic cardiomyopathy. Current guidelines lack consensus of strong recommendations for genetic counselling and testing in PPCM.^1^ This case highlights the benefits of early genetic testing for patients with PPCM, which include elucidating the mechanism of cardiomyopathy, improving understanding of disease progression, and informing risks for family members and in subsequent pregnancies.

## Description of the case

A 22-year-old primigravid woman at 40 weeks and one day gestation presented in labour and underwent Caesarean delivery for foetal indications. Her pregnancy was uncomplicated except for exertional dyspnoea, cough, and orthopnoea in the final weeks. Immediately after delivery, she suffered two pulseless electrical activity cardiac arrests. After resuscitation, she developed cardiogenic shock and was found to have biventricular heart failure with a left ventricular ejection fraction (LVEF) of 10%. She was cannulated on veno-arterial extracorporeal membrane oxygenation (VA-ECMO) and transferred to a transplant centre.

Her medical history included asthma and gastroesophageal reflux disease. Family history was notable for her maternal grandfather having suffered fatal arrhythmias at 57 years of age. She denied tobacco, alcohol, or illicit drug use.

The differential diagnosis included acute dilated cardiomyopathy exacerbation, PPCM, pulmonary embolism (PE), amniotic fluid embolism, and viral myocarditis.

Laboratory tests showed elevated N-terminal pro–B-type natriuretic peptide (1206 pg/mL) and troponin-I (0.38 ng/mL). She had normal kidney function and mildly elevated liver transaminases (alanine transaminase of 110 U/L, aspartate aminotransferase 100 U/L). She had an elevated white blood cell count (27.63 ×10^3^/µL), with an unremarkable infectious workup. Chest radiography showed an enlarged cardiac silhouette, pulmonary oedema, and small bilateral pleural effusions. Computed tomography angiogram was negative for PE. Transthoracic echocardiogram (TTE) on VA-ECMO showed an LVEF <20%, severe LV dilation, global hypokinesis, and mild right ventricular (RV) dysfunction.

She was able to be decannulated from VA-ECMO and supported with an Impella 5.5 and milrinone for RV support. Serial TTEs revealed persistent severe biventricular dysfunction, and both mechanical circulatory and inotropic support could not be weaned due to RV failure and hypotension. She had frequent premature ventricular contractions (PVCs) and runs of non-sustained ventricular tachycardia (NSVT) with right bundle branch (RBBB) morphology. Guideline-directed medical therapy was not tolerated due to symptomatic hypotension. Due to RV failure and clinical features suggesting low likelihood of LV recovery, she was felt not to be a good candidate for a durable left ventricular assist device (LVAD) and was listed for orthotopic heart transplant (OHT). She underwent uncomplicated OHT, receiving anti-thymocyte globulin for induction immunosuppression followed by standard tacrolimus, mycophenolate mofetil, and glucocorticoid maintenance therapy, and discharged on post-operative day ten.

**Figure 1:**
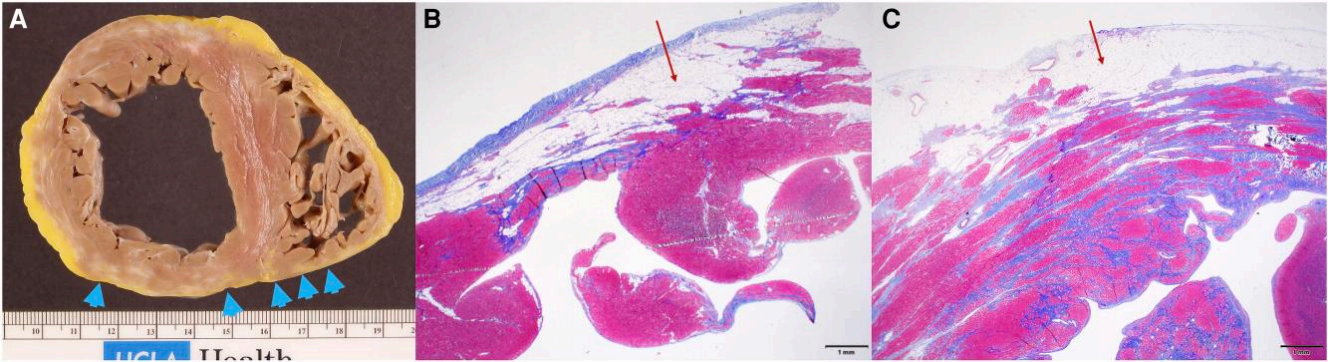
Histopathological findings of explanted heart. Pathological findings consistent with arrhythmogenic cardiomyopathy. (*A*) Gross finding of fibrofatty subepicardial infiltrate (blue arrow heads) on cross-section of explant heart. Scale bar: 1 cm. (*B*) Histologic examination of the right ventricle with Masson trichrome stain demonstrating fibrosis (blue arrow heads) with adipocyte infiltration (red thin arrows). Scale bar: 1 mm. (*C*) Histologic examination of the left ventricle with Masson trichrome stain highlighting patchy fibrofatty replacement (red arrow). Scale bar: 1 mm

**Figure 2:**
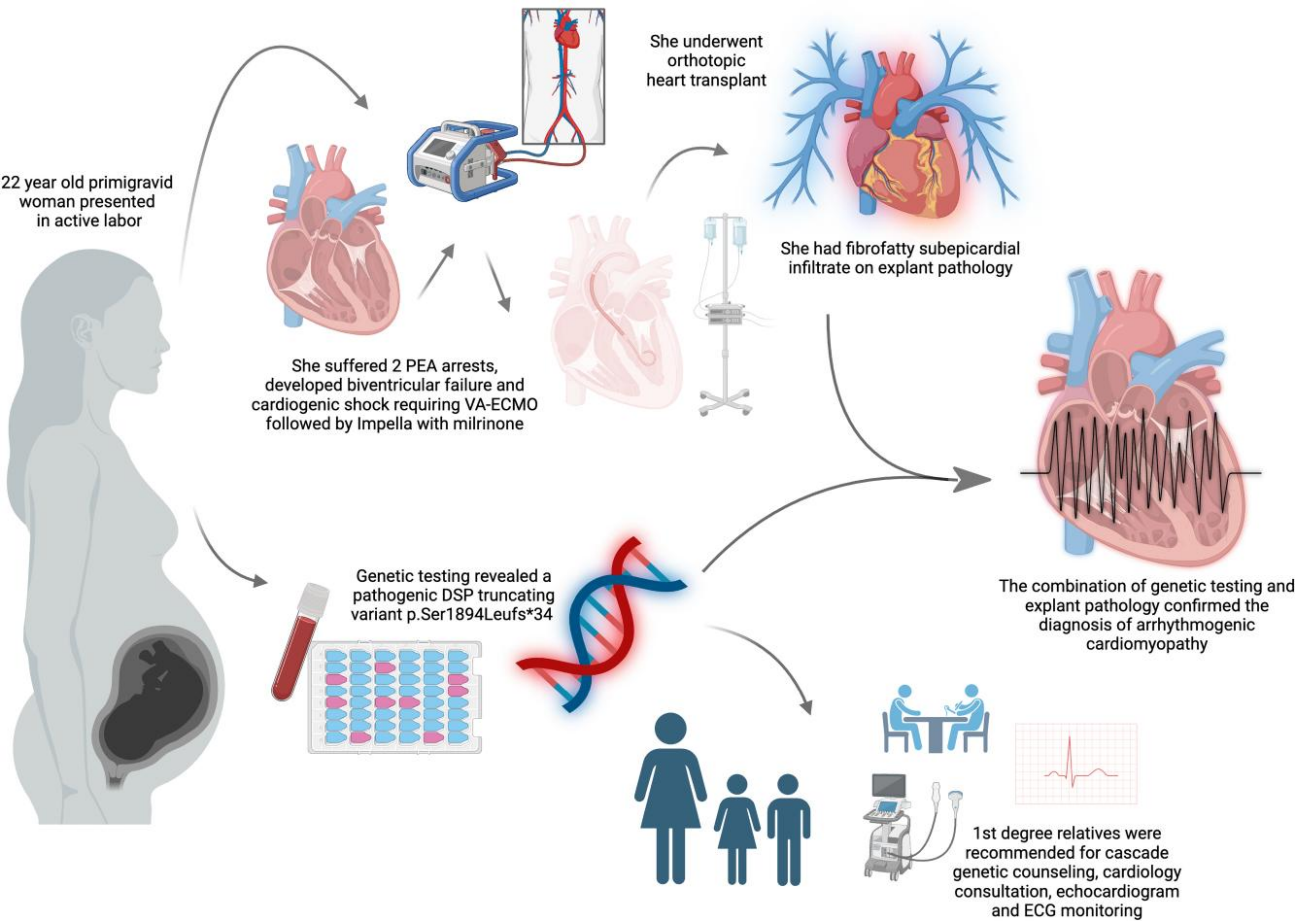
Visual summary of case. Visual summary of case illustrating the timeline of events from presentation to diagnosis. Patient presenting in labour and suffering cardiac arrest necessitating VA-ECMO, Impella and transplant. Her course is displayed demonstrating that genetic testing, counselling and explant pathology analysis co-occurred and led to the diagnosis of arrhythmogenic cardiomyopathy. PEA, pulseless electrical activity; VA-ECMO, veno-arterial extracorporeal membrane oxygenation; DSP, desmoplakin; ECG, electrocardiogram

Explant pathology revealed fibrofatty subepicardial infiltrate of both ventricles, consistent with biventricular arrhythmogenic cardiomyopathy (ACM) (*Figure 1*). Genetic testing by deletion/duplication analysis of a 100-gene arrhythmia and cardiomyopathy panel on next-generation sequencing platform was ordered through a commercial CLIA certified laboratory, identifying a heterozygous pathogenic DSP p.Ser1894Leufs*34 variant which is predicted to cause loss of function, is rare in population controls (gnomAD v4.1 0.005%), and is consistently classified by other laboratories as disease causing (ClinVar ID: 405232). Two heterozygous variants of uncertain significance were found in genes inconsistent with her phenotypic presentation, thus not clinically suspicious (CSRP3 copy number gain = 3; KCNH2 c.2696C>T (p.Thr899Met). These findings in conjunction with her hair were consistent with the clinical diagnosis of *DSP*-related biventricular ACM. DSP-mediated ACM may present with heterogeneous cutaneous phenotypes ranging from wavy/curled/coarse hair to the classic woolly hair.^2^ All her first-degree relatives were recommended for genetic counselling and cascade genetic testing; serial cardiac screening was recommended until relatives’ gene carrier status was clarified.^3^ For 2 years post-transplant, she had excellent allograft function with minimal complications. However, at month 25, she developed symptoms of heart failure and an elevated donor-derived cell-free DNA of 9.7%. Endomyocardial biopsy revealed grade 1 antibody-mediated rejection. She was admitted to the hospital for treatment but developed refractory cardiogenic shock and VT requiring VA-ECMO. She suffered embolic strokes resulting in cerebral oedema and brain death. (*Figure 2*)

## Discussion

PPCM is a diagnosis of exclusion, therefore, genetic testing should be considered for patients presenting with cardiomyopathy in the peripartum period.^4^ Patient misclassification during this period can delay management and genetic testing.^5^ The patient’s family history was significant for her maternal grandfather having died from a fatal arrhythmia at 57. However, due to her tenuous clinical condition, a cardiac MRI and genetic testing were not pursued prior to transplant. Without these data, her presentation did not initially meet Padua criteria for definite ACM, as she only satisfied one LV minor criterion (global LV systolic dysfunction) and two RV minor criteria (inverted t waves in V1/V2, and >500 PVCs in 24 hours with RBBB morphology NSVT).^6^ Her explant pathology findings met the structural RV major Padua criterion and the genetic results confirmed a diagnosis of *DSP*-related biventricular arrhythmogenic cardiomyopathy, allowing for appropriate counselling of her relatives.^7^

Although universal genetic testing is not recommended, the 2022 American Heart Failure Guidelines provide a class 2a recommendation for select patients with nonischaemic cardiomyopathy, guided by phenotype and family history.^1^ Similarly, the Heart Rhythm Society and European Heart Rhythm Association recommend considering genetic evaluation for patients with a clinical or necropsy diagnosis of ACM or reasonable suspicion of ACM. However, despite a class 2a indication for genetic testing in patients with PPCM, the European Society of Cardiology provides a class 1 indication for patients meeting diagnostic criteria for cardiomyopathy when it can inform diagnosis, treatment, and prognosis or enable cascade testing of relatives who would otherwise require long-term surveillance.^8^ Recent studies show a genetic contribution in 15–20% of PPCM cases, with prevalence likely underestimated due to undertesting.^9^ Importantly, the presence of some pathogenic gene variants associated with ACM, such as *DSP* and *FLNC*, have potential implications for management, including consideration of an implantable cardioverter-defibrillator (ICD) for prevention of sudden cardiac death.^9^ Furthermore, if these ACM-associated variants are present, they can improve prognostication, as they tend to be associated with progressive decline rather than the higher rate of LV recovery noted in others with PPCM.^1^ Knowledge of this patient’s genetic variant prior to OHT could have led to the definitive diagnosis of ACM, influenced management by providing a rationale for earlier transplant listing (rather than waiting to assess for LV recovery) or for early ICD implantation if she had stabilized. Furthermore, in cases of left-dominant ACM where RV dysfunction would not preclude durable LVAD, a genetic diagnosis could influence a decision to favour OHT in lieu of a strategy to use durable LVAD as a bridge to recovery (as is done in many cases of PPCM). Finally, if a patient with PPCM achieves LV recovery and desires subsequent pregnancy, genetic diagnoses can inform relapse risk during preconception counselling.

Importantly, there may be several barriers to implementing universal genetic counselling and testing for patients with PPCM. These include the lack of strong guideline recommendations, limited awareness about genetic testing benefits among healthcare providers, limited access to testing and counselling, and cost considerations.^10^ Finally, no large-scale data exist confirming that widespread testing improves patient outcomes, though there are several smaller published examples of benefit.^10^ Despite these drawbacks, genetic testing can serve as a useful tool in the management and counselling of patients with PPCM.

## Declarations

### Data Availability

No data were generated or analysed for this manuscript.
